# Lectures on Clinical Surgery

**Published:** 1855-06

**Authors:** James Syme

**Affiliations:** Professor of Clinical Surgery in the University of Edinburgh


LECTURES ON CLINICAL SURGERY. By JAMES SYME, Esq., Professor of Clinical Surgery in the University of Edinburgh.

LECTURE VI. *Popliteal Aneurism.*—There is a man at present in ward No. 5, with a tumor in the popliteal space, which we recognize by several symptoms, and especially by its expansive pulsation, as an aneurism. The patient is a blacksmith, thirty-two years of age. About eighteen months ago he slipped upon a wet floor, and violently stretched the parts in the left ham. He suffered pain in that situation for a week, but was afterwards unconscious of anything wrong in the leg till about six weeks ago, when he began to experience uneasiness in the ham and calf, and, on putting his hand to the popliteal space, discovered a throbbing swelling of small size, which has since rapidly increased, and is now as large as the closed fist. A violent blow or strain is almost always mentioned by the patient as the cause of popliteal aneurism. There can be little doubt that the internal coats of the vessel are injured at the time of the accident, being perhaps predisposed to rupture by disease; the aneurism then gradually forms, and increases in size, often without producing any obvious symptoms till it has attained considerable bulk, when it presses on the popliteal nerve and on the vein, causing pain, simulating rheumatism, and swelling of the foot. Even when pain does occur, it is apt to be so diffused that the attention of the patient is not drawn to the part. A cavalry officer consulted Mr. Liston in London for supposed rheumatism of the leg, and medicines suitable for that complaint were prescribed. He next consulted the late Dr. Abercrombie, who discovered a popliteal aneurism, and sent the patient to me. So little inconvenience did the tumor occasion, that, in spite of my remonstrances, this gentleman persisted in following the hounds till the day before I operated upon him. The condition of the parts in the origin of aneurism is rather a matter of inference than of direct observation. Almost the only instance on record in which the first stage of the complaint has been observed was met with in examining the body of King George II., where a crevice was found in the aorta, with a small clot external to it; and there can be little doubt that, had his Majesty lived longer, he would have been the subject of aneurism of the aorta.

Leaving now these pathological inquiries, let us consider the more important question at present, how we are to cure the disease; and, first, Is it necessary that we should interfere in the case? This leads us to speak of the prognosis of aneurism. No doubt coagulation may occur spontaneously, and lead to a natural cure. It has twice happened that a patient, having come from the country in order that I might tie the femoral artery for popliteal aneurism, within forty-eight hours of his being placed in perfect quiet, pulsation has ceased, and the tumor has subsequently disappeared. Such cases are, however, unfortunately quite exceptional. In all probability, the aneurism, if left to itself, would increase in size, and lead either to mortification from obstruction of the circulation, or to fatal hæmorrhage from rupture. We are, therefore, called upon to adopt some means of treatment, and you are aware that there are two methods for us to choose between—namely, ligature of the vessel, and the application of pressure over it. It may be said, in favor of the former, that it causes no pain whatever; since if chloroform is used, the patient does not feel the operation, and he suffers nothing after it, his only duty being to keep quiet for a fortnight or three weeks. The advantages alleged in favor of compression are, that it is safer, ligature being attended with risk of hæmorrhage or mortification of the limb, while compression is devoid of these dangers, and therefore preferable. On the other hand, if the operation of ligature be properly executed, it is almost absolutely free from danger; pressure, moreover, does not always succeed, even in the most experienced hands, and generally requires to be applied for weeks or months before any appreciable effect is produced by it, while the patient requires to be kept almost under military discipline, in the endurance of very considerable pain, as you will understand if you will apply a tourniquet on your own persons. In an institution like this, the length of time occupied is a serious consideration; for instance, a patient lately in the hospital, whose case has been published, was for seven months under treatment by compression, and at last ligature of the artery was resorted to. Here was a bed occupied for this protracted period which might have been the means of relief to many cases, with proportionate instruction to those who study here. But it may be said, in addition, that pressure is not quite so safe as it is generally stated to be; it may lead to mortification, and other disturbances, which have been described in some cases that have been published, and which, I have reason to believe, would be found more frequent if all the cases in which compression has been tried, were placed before the public. After a careful consideration of the subject, I have come to the conclusion that, if the operation of ligature be performed properly, with due attention to the niceties that render it safe, it is preferable; but if circumstances prevent this, compression should, by all means, be had recourse to. I can understand many such circumstances; for instance, a surgeon in the public service, or some remote district of the country, who has little opportunity of practising operative surgery, may feel that compression would be the safer treatment. In short, the question seems to me very similar to that of whether a catheter should be passed, or the bladder punctured, for retention of urine; if the urine can be drawn off by catheter, by all means let this be done, but if the circumstances of the case should be such that not one surgeon in a hundred could do so, it would be incumbent upon the ninety-nine to obviate the risk of rupture by puncture of the bladder; and, hence, whilst I regard the introduction of the catheter as the preferable means of relieving retention of urine, I have never undervalued puncture of the bladder as an operation in surgery. I say this the more because I read, a few days ago, in a London periodical, the statement that I "have endeavored to cast a certain amount of discredit on those who have ably devoted their time and energy to the trial of a more simple and less hazardous cure of this formidable disease."

This is a very serious accusation against any man, and particularly so when coming from one teacher of surgery against another. You, at least, gentlemen, will I hope hold me incapable of such conduct. It is indeed quite true that I have given no countenance to all the clap-trap performances of what has in recent times been denominated "conservative surgery," but it is not true that I have in any way opposed the efforts of those who have striven to improve the practice of surgery. The same lecturer says that the treatment of aneurism by compression was "in vogue," as he expresses it, "two hundred years ago," but was "abandoned after the trial of Hunter's operation in 1780." This is entirely incorrect, and for very obvious reasons, since compression, as now practised, was only thought of by following out the principle of the treatment of Hunter and Desault, (for Desault's name should always be mentioned along with Hunter's in speaking of this subject). Hunter's operation contrasted strongly in its results with the old method, which was, to cut into the aneurism and tie the vessel both immediately above and below it, a proceeding which was so rarely successful, that Dr. Wilmer, of Coventry, (1779,) says that he knew of no instance of its being so in this country. When, therefore, it was learned that the operation of Hunter was commonly followed by success, a very great sensation was produced in the medical world; but, unhappily, it soon appeared that the operation was not unaccompanied with risk of hæmorrhage, and you will find that various expedients were in consequence introduced with the hope of diminishing this danger. Instead of a simple piece of silk or fine thread, ligatures of reserve, as they were called, were also passed, to be used if necessary; thick cords or tapes were employed, a pad of lint was interposed between the vessel and the ligature the artery was exposed and forceps (*presse-artères*) were applied for a time instead of a permanent ligature, and with similar views the artery was compressed at the seat of Hunter's operation, without exposure of the vessel; and if you will look into M. Boyer's Surgery, you will find two cases recorded in which this treatment had been used successfully, and in reading the accounts it will seem to you as if you were perusing recent cases instead of those half a century old. Now I beg you to notice what took place subsequently. The cases I speak of were in 1804 and 1806; in 1807 a great step was made by the publication of a work by Dr. Jones, a graduate of Edinburgh, on the process by which hæmorrhage is arrested. He clearly made out the following important points:—that the safety of the operation of ligature is the greatest when the artery is exposed to the smallest possible extent; also, that the risk is less, the less the surrounding parts are disturbed and torn, and the more exclusively they are divided by a sharp cutting edge; further, that the ligature, which is a foreign body, should be as small as is consistent with strength, and tightly tied. It was found that if these points were attended to, the ligature of arteries was accompanied with much less risk than had previously been the case. Following out these principles, I have tied the femoral artery twenty-three times, without any bad effect. I do not by any means wish to say that every artery I happen to tie will be tied successfully, but I do say that this fact is sufficient to warrant me in maintaining that the operation, when properly performed, is not in itself a dangerous one.

Now let me explain distinctly what I mean by the operation being properly performed: I do not wish to imply something done with extraordinary dexterity, or what only a few persons can do, but I would insist upon the necessity of bestowing sufficient care upon the procedure. Take, if you please, half an hour, or a whole forenoon about it; but do not pass the needle till the artery has been sufficiently separated from the contiguous structures, to enable you to avoid all risk of injury to the vein, which is the great source of danger to be dreaded. The same surgeon whose statements I have already alluded to says, that "he has seen the artery tied quite as well by other surgeons of eminence" as it has been done by myself, "without the same beneficial effects." This is a very stupid statement—I repeat it, a very stupid statement; for the result will be a better criterion as to the performance of an operation than the opinion of a bystander.

Having been led to enter upon this subject, I will tell you of a case in which this same lecturer was acting as assistant at the tying of a femoral artery, and at which I was also asked to be present. When the dissection had been carried to a certain length,

I was asked to come forward and say whether I thought the parts were in a fit state for the needle to be passed. Not seeing the arterial coats distinctly, I said, that in my opinion, the vessel ought to be exposed more completely, remarking, that though I had never known any harm result from clearing the vessel too much, I had seen the most disastrous consequences arise from an opposite condition. These suggestions did not appear to produce any effect; the needle was passed forthwith, and the operation was supposed to be satisfactorily completed; but within a week I met the operator in the street, and was asked to be present at the dissection of the body. On making the examination, we found that the vein had been pierced by the needle, and that the man had died of phlebitis.

The patient now up stairs has been already in the hospital for ten days, during which time he has been confined to bed, and kept on rather spare diet; and as there is no reason for further delay, I intend to operate upon him to-morrow, and may at present merely remark, that the needle which I hold in my hand is, I believe, of the best form. Various complicated apparatus have been invented for tying arteries in different situations; but, with this identical needle, I have passed a ligature round nearly every artery in the body,—common iliac, external iliac, femoral, subclavian, carotid, humeral, &c., and I believe, that in all cases, it is superior to the complicated means I have alluded to. The other instruments that will be necessary, will be a knife, dissecting forceps, and hooks to hold aside the parts. As to copper knives and silver knives, which are not even yet everywhere banished from surgical armamentaria, I would recommend you to have as little to do with them as possible.

In speaking of the causes of aneurism, I forgot to mention muscular action as having a great influence upon the bloodvessels. To illustrate this effect I may mention an experiment which I once happened to see performed by the late Dr. John Reid upon a dog whose femoral artery was exposed, while the force of the blood in it was measured by a bent tube containing mercury, one end being tied in the artery; it was very striking to observe, that when the animal struggled the mercury rose in the tube, and fell again as it became quiet. This effect of muscular action on the force of the blood is of great importance, not only in the production of aneurism, but also in other matters. The greater the muscular action that occurs during an operation, the more blood will be lost; and hence the patient under chloroform will lose less blood than one who has not been subjected to its influence; the former having lost less blood, and also not suffering the pain which would otherwise attend the operation, is not liable to the faintness and collapse that almost always occurred at the close of an operation before chloroform was introduced. At that time, no other vessels being seen throwing out jets of blood, the patient was removed to bed in his collapsed state; but soon after, on the occurrence of reaction, bleeding was very apt to break out afresh; whereas at present, when chloroform is used, the vessels being tied when the circulation is in full action, secondary hæmorrhage is a comparatively rare occurrence. I am surprised that, much as has been written respecting the advantages of chloroform, this important point has not been noticed, so far as I am aware, by any writer on the subject.

[Mr. Syme tied the artery on the following day (Jan. 9th), the patient being under chloroform. The saphena vein, which was somewhat varicose, lay over the course of the vessel, and was held to the inner side during the deeper parts of the operation, which presented no difficulty. The immediate effect of the application of the ligature was to abolish pulsation in the tumor, and to produce a great diminution in its bulk. A thick worsted stocking was put upon each leg, and the limb supported in an easy position by a pillow; and, as the weight of the bed-clothes was kept from the limb by a "cradle," some cotton wadding was thrown over the leg and foot, to compensate for the difference of covering in the two legs. In the afternoon, shortly after the operation, the pulse was 64; in the evening the toes of the affected foot felt to the hand, through the stocking, slightly cooler than those of the other foot. During the night he suffered some pain in the leg. Next morning the pulse was still 64; the same difference of temperature existed between the toes of the two feet, the other parts of the feet being of equal warmth; still some pain in the leg. On the second day the pulse continued at 64, and the difference of temperature of the feet the same; the leg affected with some superficial tenderness; no swelling of the foot. On the third day the foot was in the same state, but the pulse had risen to 80; there was, however, no other evidence of constitutional disturbance. On the fourth day, the pulse was 72; the feet equal in temperature throughout; no spontaneous pain; tenderness almost gone. The wound, which was dressed at the time of the operation with interrupted sutures and dry lint, was dressed again for the first time on the evening of this day; it had united to a great extent by first intention; no blush or uneasiness about the wound. On the fifth day, pulse 60. In the evening the stocking was removed for the first time since the operation; the cuticle of the dorsum of the foot was slightly shrivelled, in consequence of the subsidence of the swelling which had existed before the operation; the tumor continued free from pulsation, and still diminishing in size; the hamstrings, instead of being pushed aside by the swelling, now lay loose beside it; general health continuing good. Seventh day: Still going on well; union by first intention has evidently been complete as regards the deeper parts of the wound, which furnish no discharge. The small superficial ulcer that remains is treated with water dressing, the patient continuing to express himself perfectly comfortable. On the 18th of Jan., Mr. Syme spoke of the case, in his lectures, as follows:—]

The man who was operated upon for popliteal aneurism nine days since is going on perfectly well; there is nothing he can complain of, except confinement to bed, which will probably not last many days longer. When you see a patient laboring under a formidable disease cured by an operation which gives him, through chloroform, no pain at the time, with the expenditure of only a few drops of blood, and little or no subsequent suffering, you naturally regard the treatment as very near perfection. Why, then, you will ask, is it not universally adopted? On account of danger to which it is said to be liable from two sources, viz: mortification and hæmorrhage. I wish to consider at present how far these two sources of danger are unavoidable, and upon what they depend. And first of mortification. This is said to be due to the limb being weakened in its vitality when the artery is tied, in consequence of the blood not flowing sufficiently freely through the anastomosing vessels, and this, it is alleged, operates more especially in small aneurisms, where there has not occurred sufficient dilatation of the anastomosing vessels to form an efficient subsidiary channel. Mortification is said to happen in two ways: in one, the weakened limb is for some time after the operation cold and impaired in sensation; then a period of reaction occurs, accompanied with pain, swelling, heat, and redness; and if this reaction be excessive, it may prove fatal to the limb by the inflammation. In the other mode of occurrence of mortification, reaction does not take place, but the limb remains cold, and its color changes to blue, its moisture exudes, and it finally becomes dry and black, like a mummy, and the death of the patient, or amputation of the limb, is the inevitable consequence. These are very frightful statements, and I confess that in the first case on which I operated I felt considerable alarm. The aneurism was of small size, and the patient (a gentleman of no great firmness of constitution originally) had been much reduced by the treatment to which he had been subjected by the late Professor Thompson and Turner, viz: confinement to bed on very low diet, with the object of promoting spontaneous cure, but with no beneficial result. To my astonishment, however, I found after the operation that there was scarcely any appreciable difference of temperature between the two limbs; that this soon passed off, and that no inconvenience of any kind was experienced. This is now, I believe, the twenty-fourth case in which I have tied the femoral artery, and I never met with any other result. There has never been any difference of temperature, except a slight coolness, which required careful examination with the hand or the thermometer to detect. There has never been any threatening of reaction, never even a partial slough, yet my patients have been old and young, (I once operated on a boy, nine years old,) and the aneurisms both small and large, as well as of recent and of long duration. I have, however, seen mortification occur in the practice of others in both the ways I have mentioned. The case with reaction was one on which Mr. Liston operated; the patient had suffered pain previously, and had been in the habit of relieving it by the use of warm baths, of which he kept an establishment. The pain continued after the operation, and the hot bathing was liberally used the same evening and next day. Swelling and reaction came on, terminating in mortification, and I assisted Mr. Liston in amputating the thigh at the middle. In other cases I have seen the limb become cool after the operation, and in the evening cold as a dead body, then blue, and finally mummified. Now there must be reasons for results such as these, and I confess that this was to me at one time a subject of very anxious inquiry; but all my own investigation would perhaps have been vain, had it not been for the observations of Mr. Guthrie, which are important on many points, but especially on the subject of injuries of arteries. He observed that the artery alone might be obstructed without death of the limb, but that this was the invariable result if both artery and vein were obstructed together.

In December, 1853, a boy, aged thirteen, employed on a railway in connexion with a colliery in Fifeshire, was riding on a wagon with his left leg hanging over one of the buffers, when the wagon came in contact with a locomotive, and the thigh was crushed above the knee between the buffers. On admission, the boy was unable to pass his urine, and I was asked to see him on account of supposed extravasation; but having drawn off some clear urine by catheter, I saw that the swelling about the perinæum and scrotum was due to effusion of blood. The femur being fractured, a long splint was applied; the limb, however, was quite cold, and afterwards changed to a blue color. When speaking in this theatre of the case, at this period, I remarked that I suspected the artery and vein had both been injured; but being desirous to give the limb a chance, I did not operate at once, as I perhaps ought to have done. By and by, however, it appeared that the limb would not recover, the toes and part of the foot became completely mummified, while the leg remained dark, and the thigh was so much disorganized, that I was obliged to amputate as high as possible below the hip-joint. On examination, we found that the popliteal artery had been torn across, and the vein much stretched and obstructed at the same place. The boy recovered slowly, and is now in good health; but his case was a very striking example of the truth of the principle alluded to. I have long been of opinion that where the results differed so remarkably, not in degree, but in kind, there must be some distinct cause for the difference, and this I believe to be some injury to the vein. I do not say always a wound ; a bruise may be sufficient to cause obstruction. In one case where mortification occurred, and where I had an opportunity of examining the limb, the needle had grazed the vein, and excited phlebitis, which had obstructed it, and of which the patient ultimately died. The only explanation that I can arrive at is, that the vein suffers somehow at the time of the operation. You are all aware that the femoral vein is intimately connected with the artery, much more so than in the case of some other vessels that may require ligature—*e. g.*, the iliac artery is but loosely connected with the vein, and great freedom or even great roughness, may be used with it without bad result. It is not so, however, with the femoral; if you do not detach the artery from the vein with the greatest nicety, you will very likely either pierce or bruise the latter. But if you do the operation in the way that I shall presently describe, you may fairly expect that, instead of mortification either with or without reaction, the only change that will occur in the limb will be a slight diminution of temperature, productive of no inconvenience, and which will disappear in a few days.

Now, we come to the other source of danger, viz: hæmorrhage; and in order to consider this, I must call your attention to the process of separation of the ligature. The first effect of the ligature is to cut through the internal and middle coats, and to draw the external coat into a small compass, not wider than a probe. The next result is the coagulation of the blood lying in the artery near the ligature, forming a conical coagulum on each side, tapering away like a worm as far as the nearest large branch. The next change is an effusion of lymph on each side of the ligature, sealing up the ends of the vessel; then a process of ulceration takes place for the separation of the foreign body, viz: the ligature, with the portion of external coat included in it. If you examine the ligature when it comes away a few days hence, you will find that it includes a substance, which is the piece of external coat that has died, in consequence of the constriction it has suffered. If the sloughing or ulceration go beyond due bounds, the lymph will be no longer able to prevent the clot from being forced out by the heart's action. The clot could do little good of itself, but it diminishes the force of the blood upon the barrier of lymph, and the longer the coagulum, the more effectual will it be in this way; hence the further the ligature is removed from a large branch, the greater will be the patient's safety. Now the femoral artery varies in its point of origin from the common femoral, and it is very important that the ligature should be applied considerably below its lowest origin; hence one great element for safety is to tie low enough, and I do not doubt that some cases of hæmorrhage have occurred in consequence of the ligature being applied too high up. I have seen many persons perform this operation at a higher point than I am in the custom of doing, which is where the artery has become overlapped by the sartorius muscle. The operation is rendered a little more difficult by the overlapping of the muscle, but this is altogether a secondary consideration. There is, however, a limit to the distance downwards; if you go beyond the sartorius, as Hunter did, you will be too near the aneurism. Whatever tends to denude the artery from its connexions will increase the extent of sloughing of the arterial coats; and whatever bruises or lacerates the surrounding textures, and so interferes with union by the first intention, will have the effect of exposing the vessel, subsequently to the operation, during the process of suppuration that will ensue. All of you ought to be aware that when an artery is exposed, so as to enter into the formation of the wall of a suppurating cavity, it is apt to give way by ulceration, whether a ligature has been applied to it or not. Arteries not unfrequently lie near abscesses; the vessel does not, however, open into the abscess till the latter has been formed into a sinus. You will ask why this is the case. It may be difficult to give a satisfactory explanation, but we must not, therefore, disregard the fact. Mr. Liston was one day making his visit at University College Hospital, London, when his attention was directed by the house surgeon to a collection of fluid in the neck of a girl. Mr. Liston, seeing that the child was in other respects unhealthy, plunged a knife into the tumor, supposing it to be an abscess, but blood, instead of matter, poured out, and it was evident that he had opened a cavity communicating with the carotid artery. Mr. Liston maintained that this was an abscess into which the vessel had opened, and applied to several persons, and amongst others, to myself, inquiring whether we had ever met with such a thing. I mentioned several cases of arteries opening into sinuses, but I believe there is no well-authenticated instance on record of an abscess communicating with a large vessel before discharging its contents.

Some time ago I was asked to see a case in this hospital, where a large pulsating and fluctuating tumor existed above the sternum. There was, however, no pulsation during inspiration, and hence, as well as from some other circumstances, I concluded that it was an abscess. I, however, declined to open it, on the ground that so long as the skin remained entire there was no risk of the great vessel opening into it; whereas, if it were opened, the artery might very probably give way into the sinus, and the patient's death would be attributed to my interference. On the following day, or the day after that, the abscess opened of itself, and discharged a large quantity of matter, then, after a day or two, there was a gush of arterial blood, and the patient died; illustrating in a very striking manner the fact of which I am speaking. Keeping these things in mind, you will see that if, through bruising of the textures adjacent to the artery with blunt instruments, union by the first intention is prevented, the vessel will form part of the wall of a sinus when the wound suppurates, and there will be risk in addition to that attending separation of the ligature, of the coats giving way, so as to produce hæmorrhage.

In performing the operation, I recommend you to use nothing but an ordinary knife, a pair of dissecting forceps, a needle for passing the ligature, and some hooks for holding the parts aside. Do not allow your assistant to draw the skin towards him while you make your incision, but, in order to keep the integument from slipping, place your hand across the thigh, and cut from it along the course of the vessel. After opening the fascia, you may perhaps be immediately upon the artery; this you may ascertain by feeling for its pulsation with your finger, which may be used for this purpose, but never for separating the textures. I think it better to divide the fascia over the sartorius, which is recognised by the direction of its fibres, when a little dissection brings you into the muscular interspace. I beg now to show you a thing, which you may think of small consequence, but which I have found very useful, viz: this form of hook for keeping the parts aside. I met with it accidentally amongst a set of old instruments, but it has proved of great service, as it not only holds the muscles to one side, but also presses them down; and in this respect the difficulty of the operation is greatly diminished by chloroform, which prevents the tension of the muscles which occurs when the patient is conscious. You will find in this, as in many surgical operations, that the result often depends upon some apparently trivial matter, on which, however, turns the whole question of a successful or fatal issue.

[The ligature separated on the 6th of February, when the cure might be considered complete.]

---

Lecture IX. Excision of the Elbow-joint, and Fracture through the Lower End of the Humerus.—You had yesterday another opportunity of seeing excision of the elbow-joint, or rather excision of the articulating extremities, which were in that case firmly anchylosed. As you have now seen two of these operations, and will probably, ere many days have elapsed, witness the third, I may make some observations at present on the subject.

Not more than twenty-five years ago, in all cases of incurable disease at the elbow, the established practice was amputation of the arm. A few cases of excision of the articulation had occurred in France, and one in Ireland by Sir Philip Crampton; but the recovery having been extremely tedious, and the limb not proving nearly equal to its natural condition, the operation had not been adopted in Great Britain. This imperfection of the results was mainly due to an error in regard to the extent of bone requiring removal. In caries, little more than the articular surface is engaged in the disease, and the part thus affected is of very limited thickness; but beyond this the bone is roughened, being covered by an effusion of osseous substance, which is not morbid any more than that thrown out to unite a fracture, or supply the loss of substance in necrosis. Moreau, father and son, were the surgeons who had performed the operation in France. Moreau says, in speaking of one of his operations, that, finding the bone roughened he cut off another inch of the shalf. Sir Philip Brampton, for a similar reason, removed the bone "three inches above the tuberosity of the inner condyle." The effect of such a proceeding is, to produce so great a relaxation of the parts, as to allow the muscles little command over the limb, while at the same time the recovery is much protracted. It occurred to me that the results might be better, if the part really diseased were alone removed. In 1827, I had under my care a young gentleman of tender years, with disease of the elbow-joint which had resisted all treatment. I wished to excise the articulation, but not finding any one in Edinburgh to sanction so unprecedented a proceeding, I reluctantly performed amputation. I had not seen him again till last year, when a tall, fine looking man entered my room, and introduced himself as the subject of this case. I confess, I felt some remorse of conscience on looking at his empty sleeve; but I had the satisfaction of reflecting that this was the last case in which I was guilty of any such unnecessary mutilation. In 1828 I performed the operation on my own responsibility, and in 1831, published a treatise on the subject, having in the interval operated on fourteen cases. This mass of evidence attracted attention, and notwithstanding very violent opposition, the operation has since become more and more generally adopted, and at last even in London, which held out longest, it has during the last few years been occasionally practised. But while thus adopted, it has shared the same fate as another operation to which I lately alluded the principles of its performance having been overlooked. There is no operation in which the mode of making the incision is of more consequence. Unless they be sufficiently free, the portions of bone cannot be removed without risk of injury to the remaining parts, and unless they be properly directed, the resulting cicatrices will interfere with the free motions of the limb; and we are not satisfied here with the result unless there be perfect flexion, extension, and rotation, with the full natural amount of strength. I believe the only incisions by which this perfect result can be obtained, are those originally practised by Moreau, which are, a transverse one, carried across the back of the articulation immediately above the olecranon, from the ulna nerve to the external condyle, and two longitudinal through the extremities of the transverse, so that the incision altogether has the form of the letter H. When the flaps have been raised, free access is gained to the articulation, and when the operation is completed, the edges of the transverse incision, if brought accurately together, generally adhere by first intention, instead of healing by granulation and adhering to the bone, so as to prevent free motion. Having dissected up the flaps, do not attempt to remove all the articulation at once, as otherwise there will be a risk of cutting the ulnar nerve, with consequent deficiency of sensation and atrophy of the limb; but, having exposed the olecranon, cut it off in the first instance, so as to get free access to the joint, then divide the external lateral ligament, and having pushed the ulnar nerve over the inner condyle free the end of the humerus and saw it off on a line with the tuberosities; and, lastly, move in succession the ends of the radius and ulna on a line with the base of the coronoid process. More than the extent thus defined would be unnecessary and injurious, while less would hardly remove the disease, and, even if it did, would incur the risk of anchylosis. In the cases you have happened to see there has been anchylosis, resulting in one from disease, in the other from injury, and therefore there was some deviation from the ordinary plan of proceeding, but its general principles were kept in view.

The operation of excision of the elbow was not employed for anchylosis till long after it had been adopted for disease, since I had operated more than a hundred times for the latter before I did so for the former. Though often requested to excise the joint under these circumstances, I had not been prepared to interfere with the healthy integuments merely for the convenience of the patient. But at last a young man, from the Highlands of Perthshire, came to consult me with both arms permanently extended, one immovably so, and the other admitting of but a very slight degree of motion at the elbow; resulting, in the one case, from fracture with dislocation, in the other from dislocation only. It is impossible to imagine a greater degree of helplessness than this young man's, who was obliged to seek assistance in the performance of the most ordinary offices. The case called loudly for interference, and I performed excision of one elbow. The result was an arm so perfect that it could not be distinguished from a sound one till his sleeve was removed, exciting some surprise at the Medico-Chirurgical Society of this city, where I exhibited him several years ago. Encouraged by this success, I have since repeatedly excised the elbow for anchylosis; and I have been rather amused at a feeble attempt lately made in London to claim the credit due for this application of the operation for those who have done it subsequently.

I have now to direct your attention to another set of cases of great interest.

In one of the early days of November, a boy came here, as you may remember, with one of his arms fixed in the extended posture, in consequence of an accident that had happened three or four weeks previously, causing a fracture of the humerus, simulating dislocation. Before puberty, a fall on the hand or elbow, even without any great degree of violence, is apt to cause fracture of the lower end of the humerus, which may occur in various directions, being in some cases a separation of the epiphysis, in others an oblique fracture through the condyles; and the resistance to the action of the triceps being removed, such an injury assumes the appearance of dislocation of the bones of the forearm backwards. A surgeon is sent for and recognises the features of a dislocation; he feels a dull grating on attempting to restore the bones to their natural position, and, having reduced the dislocation, as he supposes, contents himself with ordering rest and warm fomentations. Very speedily the weight of the limb, and the action of the muscles, reproduce the displacement; the surgeon, perhaps, never sees the case again, or if he does, attributes the deformity to swelling consequent on the injury. When, however, the swelling has subsided, the arm remains not only distorted, but stiff, and the patient is a cripple for life. I have known more professional reputations suffer from this error than from any other in practical surgery that I could mention. All the treatment that is required for this injury is keeping the arm in a sling at a right angle, with the elbow supported by a figure of 8 bandage, with compresses of lint before and behind. Since this case, although the month (November) is not yet done, we have had three others of a similar nature, proving the frequency, not only of the injury, but also of its erroneous treatment. In the first of these cases, as only three or four weeks had elapsed since the accident, I hoped something might be done by forcible flexion of the limb, and chloroform having been administered, I succeeded in bending it to an acute angle. So far, the gravity of the case is already much diminished, for it is a great thing to have the arm bent rather than extended, even should it remain stiff. I remember a horse dealer from Ireland, who came to me with one of his arms fixed at an obtuse angle, and told me he would give me the best horse in his possession if I could bring his arm into such a form that he could put a fork to his mouth. I then declined interference, but if he were to apply now, I should have no hesitation in performing excision of the elbow. But I hope that mobility also may be attained in the case just referred to.

The second case was of fifteen weeks' standing, and, as any forcible attempts to bend the arm were out of the question at this period, the case was dismissed as hopeless except through an operation.

The third case is also of fifteen weeks' standing, and the boy, who is thirteen years of age, is now before you. The accident occurred in the following way—he fell on his right hand, with the arm bent, and while it remained in this position another boy was thrown upon it. He applied to a bone-setter, who told him his arm was dislocated; and, having attempted reduction, dismissed him with the arm at a right angle. He afterwards went to a regular medical practitioner, who said the bone had been splintered as well as dislocated; but that it was too late for remedy. You see the olecranon is extremely prominent—almost like the os calcis; while the end of the humerus is to be felt too far forwards. These are the characters of dislocation; but a thickening about the humerus shows that there has been something more. This boy has been sent from Lanark for the purpose of having excision of the elbow-joint performed; and I, therefore, intend to do it, my only doubt being whether an attempt should not first be made to bend the arm after subcutaneous division of the tendon of the triceps. I fear, however, that the prominence of the end of the humerus forwards is too great to admit of bending of the limb without removal of the ends of the bones; but this I shall consider further. This boy, J. B——, constitutes the fourth case. He is thirteen years old, and says that six weeks ago he fell from a wall upon his left hand. He went to a surgeon, who discovered fracture of the external condyle of the humerus. And the case seems to have been very properly treated in the first instance; but, probably from some negligence in the after treatment, the arm subsequently became extended and stiff, as you see. But the deformity is not nearly so great as in the last case. Here, as in the first of the series, I shall try what can be done by forcible bending of the limb.

[Chloroform was now administered; after which Mr. Syme both bent and extended the arm to nearly the full degree. In the afternoon, it could be moved through a considerable angle without pain; and the boy afterwards left the hospital in a greatly improved condition. Mr. Syme having come to the conclusion that resection of the joint afforded the only prospect of improvement to the limb, in the third case mentioned, performed the operation on the 6th of December. Having made the incisions in the usual way, dissected up the flaps, and removed the olecranon with bone-pliers, he found it necessary to deviate from the usual course, which is to take off next the end of the humerus, as the radius and ulna, being dislocated backwards, overlapped it, and were, therefore, removed in the first instance. The end of the humerus was of a very peculiar form in consequence of the articulating part below the condyles having been broken off, and displaced forwards, and then united to the rest of the bone. Having sawn off the extremity of the humerus, just above the condyles, Mr.

Syme brought together the edges of the incisions with interrupted suture, taking particular care to retain the margins of the transverse incision in accurate apposition. The arm being then carefully held half bent in the position it was intended to maintain for the next few days, Mr. Syme placed thick pieces of folded lint upon the elbow, so arranged as to leave parts of the line of incision free for the escape of discharge, and then applied a bandage, six yards in length, in the figure-of-8 form, to support the limb. Next day, Mr. Syme exhibited the ends of the bones to the class, and made the following remarks:—]

I am now able to show you the portions of bone which were removed in the operation yesterday, placed in their relative position. You see that a prominence like an exostosis projects forwards from the lower end of the humerus; this is the articular part below the condyles, which has been separated from the rest, and displaced forwards; the articular surface is covered, you observe, but very imperfectly, by cartilage, and has evidently not been used for a long period as part of a joint. The ends of the radius and ulna, which were once articulated with it, are displaced backwards, the tip of the coronoid process resting in the posterior hollow of the humerus, and the olecranon projecting proportionately backwards. The operation was conducted essentially after the method which I have described to you; but owing to the unusual form of the bones, in addition to the fibrous union that existed between them, the proceeding was far more difficult than it is in ordinary cases of disease, or even of anchylosis, and I believe that no other incision than that of the H form would have enabled me to obtain sufficiently free access to the parts. I lately strongly recommended this form of incision to you and told you that it is what Moreau originally used, and what I have always practised; and you may therefore imagine that I was much surprised to find, in a work which has been sent to me by its author, M. Guerin, giving the latest Parisian views upon operative surgery, (for it is dated 1855,) the statement that I make use of the simple longitudinal incision, which I consider to be the most objectionable of all. This shows how careful you should be in accepting such statements; and I may here remark that the surgery of Paris, which a hundred years ago was, without doubt, the best in Europe, is at present characterized by frivolity, pretension, and total disregard of practical utility.

[Everything went on favorably, the arm being dressed at intervals of two days with pads of dry lint, and a figure-of-8 bandage, as at the time of the operation; the stitches being removed as they became loose by ulceration, and strips of adhesive plaster substituted till union had become firm. The method of dressing, which is that always employed by Mr. Syme for these cases, unless there be some special circumstance to contraindicate it, necessarily involved some slight movement of the elbow from the first; but after about ten days some degree of passive motion was purposely given, in order to insure mobility of the elbow. On the 8th of July, little more than four weeks after the operation, Mr. Syme showed the boy to the class. At that time the elbow was almost completely healed; the arm could be bent and extended nearly to the full extent, and passive pronation and supination were almost perfect; the boy could also lift considerable weights with the arm, but was discouraged from making such attempts for the present. He left the hospital on the 1st of February; at that time the elbow was completely healed. Passive motion of the limb could be performed to the full extent, and he could also bend and extend the arm through a small angle without the assistance of the other hand, and had very considerable power of pronation and supination; the arm was still gaining strength, and within the last few days he had been able to carry a large tea-kettle full of water in that hand. Mr. Syme, in speaking of the case in his lecture, said:—]

It has often been a question—What is the condition of the parts after excision of the elbow-joint has been performed, and the arm has recovered its powers of motion? Supposing the state of things to be the same as in an ununited fracture, there would be merely a mass of ligamentous texture between the bones. With regard to this point, I would refer you to a probationary essay written on the subject of Ununited Fracture by my old friend Dr. Sharpey, of London, when a candidate for the fellowship of the College here. I had myself, till within the last few days, had only one opportunity of examining a limb after excision of the elbow; in that case, there was only ligamentous union, but the examination was made within one year of the operation; but I am now in a position to show you the condition of matters at a much later period, which I regard as of extraordinary interest. The patient was admitted into the hospital, under the care of Dr. Dunsmure, on account of a railway injury, of which he died, and the following account of him while in the hospital has been furnished me by Dr. David Christison, house-surgeon to Dr. Dunsmure:—

"A. E——, aged thirty-eight, was admitted on the 17th of December. He had sustained a compound fracture of both legs: one so severe that amputation of the thigh was at once performed. He died about eight days afterwards. He was a tall, very muscular man, weighing fourteen stone. About nine years ago, he was recommended by some surgeons to lose his left arm, on account of disease of the elbow-joint. This was in Aberdeen; but he resolved, before submitting to amputation, to consult Mr. Syme. He accordingly came to Edinburgh in 1846, when excision of the elbow-joint was performed in this hospital by Mr. Syme.

The patient stated that he made a rapid recovery, and had since been employed as a guard on the Edinburgh and Glasgow Railway. When questioned as to the use of his arm, he always made the same reply, that he knew no difference between it and the other limb, that he could lift an equal weight with them, and that in swinging himself about from one carriage to another, when the train was in motion, and such other dangerous proceedings, he trusted one limb quite as much as the other. On accurately comparing the mobility of the two joints, one could observe that the extent of motion was not so great on the side where excision had been performed as on the other; but flexion, extension, pronation, and supination were as perfect as could be desired for all useful purposes. The external aspect of the joint was wonderfully natural, the most obvious deficiency being the absence of the olecranon. The limb was not quite so muscular as that of the opposite side."

The ends of the bones concerned in the elbow were removed after death; they have been since dissected carefully, and are now before you.

"The elbow has a very remarkable appearance; the ends of the bones concerned, though quite different in shape from the normal condition, being yet so adapted to each other as to form a secure hinge-joint. The ulna is devoid of either olecranon or coronoid process, and instead of embracing the end of the humerus, is received, along with the radius, into the forked end of the os humeri. This form of the last-named bone appears to have been produced by the growth downwards of the two processes which appear to be entirely new formations. The ulna is separated a short distance from the humerus, the interval being occupied by a rather lax and vascular ligamentous union, of which a portion has been left as a specimen; but the radius forms, with the humerus, a true articulation, lubricated with synovia; the surface of the radius, where it touches the humerus, is rounded, the corresponding concave part of the humerus fitting accurately to it; these surfaces are in part covered with fibro-cartilage, and partly bare, and of porcellaneous hardness; but it is interesting to observe that a bare part of one bone is always opposed by a cushion of fibro-cartilage upon the other. The radius and ulna are also adapted to each other; the tuberosity of the radius, into which the tendon of the biceps is inserted, being received into a concavity of the ulna, formed in part by a growth of the latter bone, upwards, into a process that curves over the rounded end of the radius, and gives attachment both in front and behind to a strong and distinct orbicular ligament, which is but loosely connected with the radius, and allows of its free play in rotation. To compensate for loss of the head of the radius, which was removed in the operation, a prominent lip has grown out around the upper margin of the bone at its outer part, which thus has a purchase on the orbicular ligament, like the natural head. Between the radius and ulna there is partly loose ligamentous union, and partly a true articulation, lined with synovial membrane; the expanded insertion of the tendon of the biceps serving to cover the articulating part of the radius, while the depression on the ulna is lined with the nearest approach to pure cartilage that is to be found in this elbow. To the naked eye it looks quite like cartilage; and under the microscope differs from it only in the fact that the matrix is obscurely fibrous. Two strong lateral ligaments join the extremities of the processes of the humerus to the orbicular ligament on the one side, and to the ulna on the other; a separate piece of bone (sesamoid) is embedded in the internal lateral ligament at its juncture with the ulna. The extremity of the prolongation of the ulna, upwards, is also a separate piece united to the rest by ligament. The anterior ligament of the joint has been cut away; it was extremely strong, so as to compensate for the absence of the olecranon in checking backward movement of the forearm beyond the limit of complete extension; it was attached above to the anterior surface of the humerus, and below to the upper edge of the orbicular ligament, and the anterior surface of the ulna; there was also a strong posterior ligament. It will be observed that there has been in this elbow a great growth of bone as well as of ligament. As Mr. Syme remarked, it would appear as if the humerus had labored under the delusion that it was to unite with the other bones, and material had been thrown out, as in a fracture, adapted for forming osseous union; but, as in an ununited fracture, the motion of the parts has prevented the formation of bone, except in some directions, the union being elsewhere ligamentous, and in some parts the prolonged free use of the joint has led to the formation of a true articulation, with accurately adapted osseous surfaces, either covered with fibro-cartilage, or smoothed over by porcellaneous deposit, and lubricated with synovia."

